# Stoichiometry and Morphology Analysis of Thermally Deposited V_2_O_5−x_ Thin Films for Si/V_2_O_5−x_ Heterojunction Solar Cell Applications

**DOI:** 10.3390/ma15155243

**Published:** 2022-07-29

**Authors:** Gwan Seung Jeong, Yoon-Chae Jung, Na Yeon Park, Young-Jin Yu, Jin Hee Lee, Jung Hwa Seo, Jea-Young Choi

**Affiliations:** 1 Department of Metallurgical Engineering, Dong-A University, Busan 49315, Korea; wjdrhkstmd12@gmail.com (G.S.J.); dbsco0306@naver.com (Y.-C.J.); nayeon2385@gmail.com (N.Y.P.); tq6284@naver.com (Y.-J.Y.); 2 Department of Chemical Engineering, Dong-A University, Busan 49315, Korea; dljh82@gmail.com; 3 Department of Physics, University of Seoul, Seoul 02504, Korea; seojh@uos.ac.kr; 4 Department of Materials Sciences & Engineering, Dong-A University, Busan 49315, Korea

**Keywords:** transition metal oxide, vanadium oxide, passivation, heterojunction solar cell

## Abstract

In recent decades, dopant-free Si-based solar cells with a transition metal oxide layer have gained noticeable research interest as promising candidates for next-generation solar cells with both low manufacturing cost and high power conversion efficiency. Here, we report the effect of the substrate temperature for the deposition of vanadium oxide (V_2_O_5−x_, 0 ≤ X ≤ 5) thin films (TFs) for enhanced Si surface passivation. The effectiveness of SiO_x_ formation at the Si/V_2_O_5−x_ interface for Si surface passivation was investigated by comparing the results of minority carrier lifetime measurements, X-ray photoelectron spectroscopy, and atomic force microscopy. We successfully demonstrated that the deposition temperature of V_2_O_5−x_ has a decisive effect on the surface passivation performance. The results confirmed that the aspect ratio of the V_2_O_5−x_ islands that are initially deposited is a crucial factor to facilitate the transport of oxygen atoms originating from the V_2_O_5−x_ being deposited to the Si surface. In addition, the stoichiometry of V_2_O_5−x_ TFs can be notably altered by substrate temperature during deposition. As a result, experimentation with the fabricated Si/V_2_O_5−x_ heterojunction solar cells confirmed that the power conversion efficiency is the highest at a V_2_O_5−x_ deposition temperature of 75 °C.

## 1. Introduction

Crystalline Si (c-Si) solar cells are considered to be promising next-generation energy providers as one of the most mature technologies in the renewable energy market. However, from an economic viewpoint, c-Si solar cells are still inferior to existing energy sources based on fossil fuel. Consequently, much work remains to be done to drastically increase their market share, keeping in mind the pressing issue of global warming. The low economic feasibility of existing c-Si solar cells is primarily attributable to following factors: (1) their excessive consumption of materials (e.g., Si) and (2) relatively complicated and high-temperature fabrication process. Therefore, various cost-saving technologies have recently been explored. One representative technology is the production of solar cells using a thin Si wafer (<50 µm) to reduce the required amount of Si (i.e., the material that accounts for more than 40% of the cost of manufacturing a solar cell module) [1]. This technology is able to considerably lower the cost of materials for solar cell production. However, because Si has a low absorption coefficient in the long-wavelength region [2,3], the amount of light absorbed by a Si absorber with a reduced thickness also decreases. This, in turn, lowers the power conversion efficiency (PCE) of the solar cells [4,5]. Attempts to address this shortcoming have resulted in the exploration of various technologies to improve the productivity while simplifying the manufacturing process. A possible approach to overcome these problems includes the introduction of novel materials which would enable the development of low-cost and high-efficiency solar cells after combining with c-Si. Thanks to these efforts, many novel materials with distinct optical and electrical characteristics such as perovskite [6,7], organic polymers [8,9], transparent conductive oxides [10], and transition metal oxides (TMOs) [11,12] have been developed. Recently, many researchers also actively investigated the realistic application of these materials to fabricate c-Si-based heterojunction solar cells (Si-HSCs), which could minimize the cost issue stemming from complicated and high-temperature fabrication processes such as the thermally diffused doping process to build a PN junction in a conventional c-Si-based solar cell [13,14].

**Figure 1:**
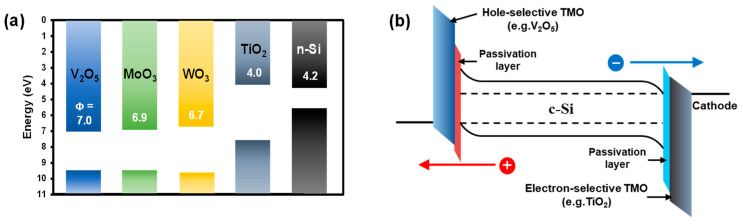
(**a**) Reported work functions (Φ) for TMOs (V_2_O_5_, MoO_3_, WO_3_, and TiO_2_) and (**b**) energy band diagram with electron- and hole-selective contacts for c-Si/TMO HSCs.

Among them, TMOs (e.g., V_2_O_5_, MoO_3_, WO_3_, and TiO_2_) are useful as excellent carrier-selective hetero-contact materials for n-type and p-type Si, both of which have work functions with a wide energy range (Φ_TMO_ = 3~7 eV), as shown in Figure 1 [15,16]. They also offer excellent window layer characteristics that can minimize the decrease in PCE resulting from the parasitic absorption by the front layers of solar cells because of their large energy band gap (E_g_ > 3 eV) [17,18,19]. In addition, TMOs are promising for reducing the processing cost, as the layers can be formed via low-cost processes such as evaporation (e.g., thermal and electron-beam) or spin coating [20,21,22]. Recently, a high PCE of 22.5% was achieved using a Si/TMO HSC in which TMOs (e.g., V_2_O_5_, MoO_3_, and WO_3_) were employed as the hole-selective contact layer. This promising achievement demonstrates the high potential of Si/TMO HSCs as low-cost next-generation solar cells capable of yielding high efficiency [23].

Despite the aforementioned advantages, the carrier recombination velocity at the Si/TMO interface created by depositing the TMOs is generally high. This situation arises because of the incomplete formation of the silicon dioxide (SiO_2_) layer, which acts as a passivation layer for the Si surface, at the low temperatures at which the TMOs are processed. The resulting short lifetime of the photo-generated carriers lowers the PCE of the solar cell [24]. The development of a method that would enable the formation of high-quality passivation layers for the Si surface in the form of a TMO deposition is, therefore, essential to fabricate high-efficiency Si/TMO HSCs.

In this study, we firstly investigated the deposition temperature effect of TMO, specifically vanadium oxide (V_2_O_5−x_), to form a SiO_y_ (0 ≤ y ≤ 2) passivation layer for Si surfaces. In many previous reports, the passivation effect of TMO for Si surfaces was studied, but they mostly focused on comparisons between different kinds of TMO such as V_2_O_5_, MoO_3_, and WO_3_, or high-temperature post-thermal annealing effects after TMO deposition [25,26]. In this report, V_2_O_5−x_ was deposited at various Si substrate temperatures below 150 °C. Subsequently, the (1) stoichiometry, (2) morphology, and (3) Si surface passivation capability of the deposited V_2_O_5−x_ thin films (TFs) were investigated. Our results clearly revealed that the passivation characteristics of the Si surface could be noticeably improved by adjusting the temperature of the Si substrate, i.e., the deposition temperature, but that the temperature had to remain below 100 °C for the thermal deposition of V_2_O_5−x_.

The effect of the V_2_O_5−x_ deposition temperature on the PCE of the Si/V_2_O_5−x_ HSC was evaluated by fabricating Si/V_2_O_5−x_ HSCs at different substrate temperatures. The HSC with the most enhanced PCE was fabricated at a deposition temperature of 75 °C, and this PCE was 25% higher than that of the sample that was not heated during the deposition. These results demonstrated that high-efficiency Si/TMO HSCs could potentially be fabricated by simply making a minor adjustment to the process temperature for TMO deposition.

## 2. Experimental

### 2.1. Materials and Sample Preparation

A double-sided polished n-type CZ silicon wafer with a (100) orientation, thickness of 280 µm, and resistivity of 1.7–2.3 Ω-cm was cut to a size of 2 × 2 cm. The wafer was cleaned by sequential ultra-sonication with acetone, methanol, and distilled water (DI water) for 15 min each. Subsequent standard RCA cleaning with a mixture of NH_4_OH, H_2_O_2_, and DI water at a volume ratio of 1:1:5 served to remove residual organic contaminants from the wafer surface. The native oxides on the Si surface were removed by immersion in dilute HF (1 vol.%) for 1 min. The V_2_O_5−x_ TF (V_2_O_5_ powder, 99.99%, Sigma Aldrich, St. Louis, MI, USA) was thermally deposited at 0.2 Å/s at various substrate temperatures (no heating, 50, 75, 100, and 125 °C) at a vacuum level of ~1.0 × 10^−6^ mbar.

Four different sample structures were fabricated in this study. The first was a V_2_O_5−x_ (15 nm)/n-Si/V_2_O_5−x_ (15 nm) sample structure for measuring the minority carrier lifetime ($\tau_{eff}$); the second was a Ag (200 nm)/V_2_O_5−x_ (15 nm)/n-Si structure with grid contact for measuring the sheet resistance via the transmission line method (TLM); the third was a V_2_O_5−x_ (5 nm and 15 nm)/n-Si structure for analyzing the surface morphology and XPS characteristics of the V_2_O_5−x_ TF; and the fourth was a Ag (200 nm)/V_2_O_5−x_ (15 nm)/n-Si/Al (200 nm) structure for measuring the built-in potential (V_bi_).

### 2.2. Characterization

The dependence of $\tau_{eff}$ on the V_2_O_5−x_ deposition temperature was analyzed by measuring the $\tau_{eff}$ with a photo-conductance decay system (WCT-120, Sinton Instrument Inc., Boulder, CO, USA). The surface morphology of the deposited V_2_O_5−x_ TF was examined by acquiring atomic force microscopy (AFM) images in the tapping mode (Veeco Multi-Mode-V). The changes in the compositions of the V_2_O_5−x_ and the formed SiO_x_ layers arising from oxidation and reduction reactions between the deposited V_2_O_5−x_ TF and the Si surface were studied using X-ray photoelectron spectroscopy (XPS, ESCALAB 250XI, Thermo-Fisher, Waltham, MA, USA). The conductivity of the V_2_O_5−x_ TF was derived using the sheet resistance measured via the TLM. V_bi_ was obtained by measuring the capacitance–voltage (C–V) relationship using the Agilent E4980A LCR meter. Finally, the PCE of the fabricated HSCs was measured under the simulated air mass (AM) 1.5G condition after calibration using a standard silicon reference cell.

## 3. Results and Discussion

**Figure 2:**
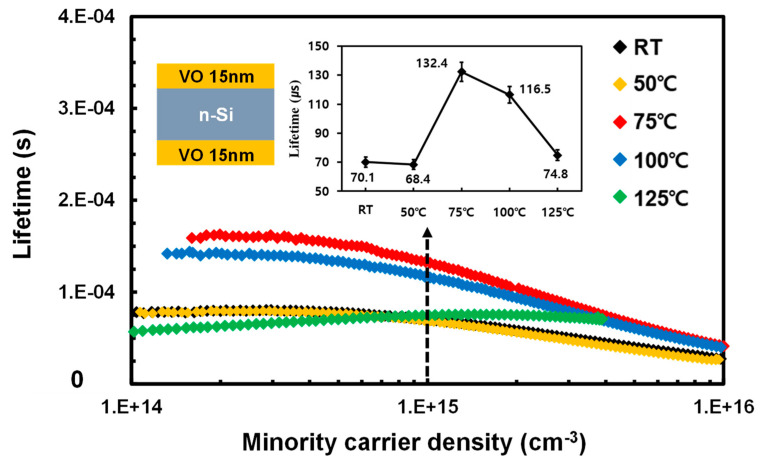
Minority carrier lifetimes plotted against the minority carrier density. Carrier lifetimes were measured at different V_2_O_5−x_ deposition temperatures for the cell structure in the inset. (Note: The vertical black dashed arrow indicates the carrier injection level of 1.5 × 10^15^ cm^−3^ at which the $\tau_{eff}$ values in the inset were derived).

In preparation for the $\tau_{eff}$ measurements to assess the effect of the V_2_O_5−x_ deposition temperature on the Si surface passivation, samples were fabricated as a sandwich structure by depositing V_2_O_5−x_ on both sides of the Si substrate at the following deposition temperatures: RT (i.e., no heating), 50, 75, 100, and 125 °C. The results are shown in Figure 2.

As shown in Figure 2, the $\tau_{eff}$ values of the samples measured at RT and 50 °C were comparable at 70 μs and 68 μs, respectively. However, at 75 °C, the $\tau_{eff}$ dramatically increased to 132 μs. In contrast, the samples at 100 °C and 125 °C had $\tau_{eff}$ values of 116 μs and 75 μs, respectively, indicating a decreasing trend at deposition temperatures beyond 75 °C. This result implies that the passivation effect of the V_2_O_5−x_ TF can be altered by adjusting the deposition temperature, and that effective passivation was possible even at temperatures below 100 °C. Most importantly, this result reveals the existence of a specific deposition temperature at which the passivation effect is significantly enhanced. The improved Si surface passivation effect is expected to lead to a high short-circuit current (J_SC_) and open-circuit voltage (V_OC_), by decreasing the reverse saturation current (J_0_) when actual Si/TMO HSCs are fabricated [27]. According to a previous study, the Si surface passivation effect of a deposited V_2_O_5−x_ TF arises from the formation of a SiO_x_ layer at the V_2_O_5−x_/Si interface when the oxygen atoms in V_2_O_5−x_ migrate to the Si surface during V_2_O_5−x_ deposition [28]. This migration of oxygen (O) atoms to the Si surface originates from the more negative Gibbs formation energy (ΔG) for SiO_2_ compared to that of V_2_O_5−x_. This is evident from the following chemical formula and the change in the Gibbs free energy ($\Delta G_{Si-O_{2}}$) [25,29].

$$\frac{5}{2}\mathrm{Si}\;+\frac{5}{2}O_{2}\;\to \;\frac{5}{2}{\mathrm{SiO}}_{2}\;\;\;\;\;\;\;\;\;\;\;\;\;\;\;\;\;\;\;\Delta G_{Si-O_{2}}=-858\;\mathrm{kJ}/\mathrm{mol}\;$$

$$2V\;+\frac{\;5}{2}O_{2}\;\to V_{2}O_{5}\;\;\;\;\;\;\;\;\;\;\;\;\;\;\;\Delta G_{V-O_{2}}=-573\;\mathrm{kJ}/\mathrm{mol}\;$$

Therefore, when V_2_O_5−x_ is deposited on the Si surface, the migration of the O atoms in the V_2_O_5−x_ TF to the Si surface for SiO_2_ formation is induced by the negative $\Delta G_{Si-V_{2}O_{5}}$ (−285 kJ/mol), as shown below.

$$\frac{5}{2}\mathrm{Si}\;+{\;V}_{2}O_{5}\;\to \;\frac{5}{2}{\mathrm{SiO}}_{2}+2V$$

$$\;\Delta G_{Si-V_{2}O_{5}}=\Delta G_{Si-O_{2}}-\Delta G_{V-O_{2}}=-285\;\mathrm{kJ}/\mathrm{mol}\;$$

**Figure 3:**
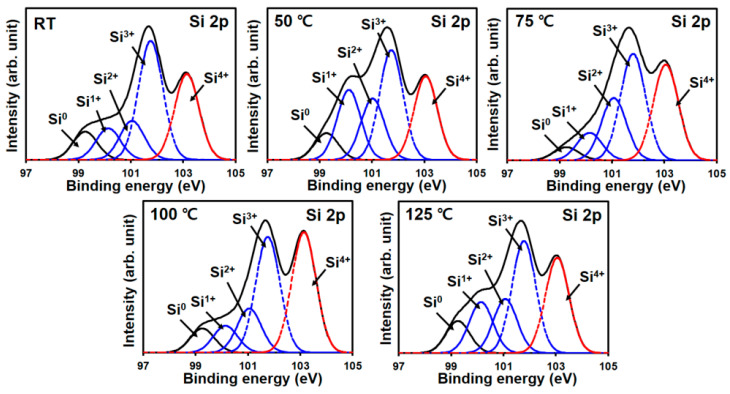
Si 2p spectra of SiO_y_ formed at the V_2_O_5−x_ (15 nm)/Si interfaces at each of the different temperatures.

**Figure 4:**
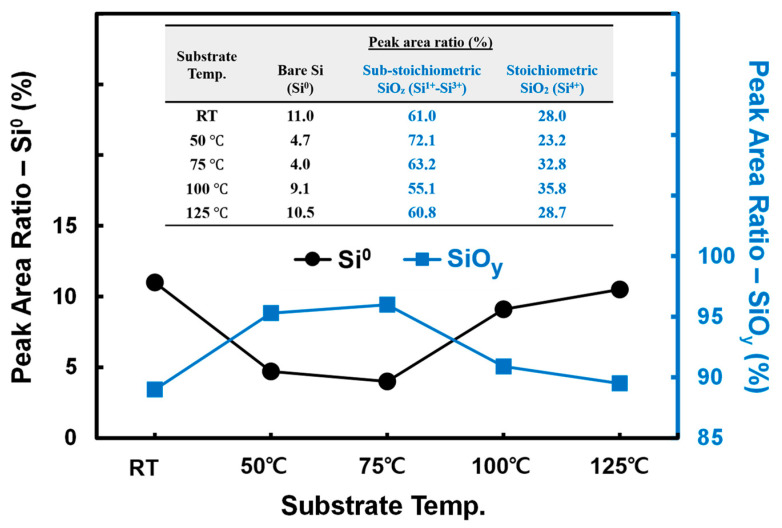
Peak area ratios of Si states calculated by measured Si 2p doublet spectra.

However, the spontaneity of this thermodynamic reaction would be expected to increase when the temperature increases [30]. This led us to predict that, in our study, an increase in the V_2_O_5−x_ deposition temperature would lead to a gradual increase in $\tau_{eff}$ owing to the formation of SiO_y_ (0 ≤ y ≤ 2) on the Si surface. However, as shown in Figure 2, $\tau_{eff}$ increased until 75 °C, above which it decreased again at the higher temperatures of 100 °C and 125 °C. Therefore, the XPS profiles of these samples were measured to study the effect of the deposition temperature on the formation of the SiO_y_ passivation layer at the V_2_O_5−x_/Si interface. The Si 2p XPS profiles, shown in Figure 3, are the Gaussian profiles, which were deconvoluted to reveal peaks for the Si substrate (Si^0^, 99.2 eV), sub-stoichiometric SiO_z_ (Si^1+^~100.15 eV, Si^2+^~101.05 eV, Si^3+^~101.75 eV), and stoichiometric SiO_2_ (Si^4+^~103.15 eV) [31,32]. This enabled the ratios of Si^0^, SiO_z_, and SiO_2_ at the Si/V_2_O_5−x_ interface to be determined according to the deposition temperature by integrating the area of each separated peak. The ratio change between Si^0^ and SiO_y_ with deposition temperature is presented in Figure 4; the detail ratios are also listed in the embedded table.

As is evident from the embedded table in Figure 4, when the temperature was increased from RT to 75 °C, the ratio of Si^0^ decreased from 11% to 4%, but those of SiO_z_ and SiO_2_ increased. The ratio of SiO_2_ (i.e., Si^4+^) increased particularly at 75 °C, confirming the formation of the highest quality SiO_y_ passivation layer at 75 °C compared to those at RT and 50 °C. However, the increase in the SiO_2_ ratio is small compared to that at 75 °C, whereas the increase in the Si^0^ ratio is observed to be significant; consequently, at 100 °C, the total SiO_y_ (i.e., sum ratio of SiO_z~1.5_ and SiO_2_) formation ratio decreased as shown in Figure 4. At 125 °C, the further decrease in the total SiO_y_ ratio is mainly attributed to the reduced ratio of SiO_2_.

Based on the Si 2p XPS profiles, the deposition temperature has a marked influence on the V_2_O_5−x_ passivation effect, even below 100 °C. However, as was previously reported, TMOs are well known to require relatively high deposition temperatures (>400 °C) to thermally induce the effective reduction of the TMOs to form SiO_y_ on the Si surface [26,33]. Therefore, we assume that the enhanced passivation effect we observed in our work at such a low temperature most probably had morphological origins rather than the TMO reduction being facilitated by temperature. To confirm our assumption, the initially deposited 5 nm thick V2O_5−x_ layer was analyzed by AFM to study the morphological change at different deposition temperatures.

**Figure 5:**
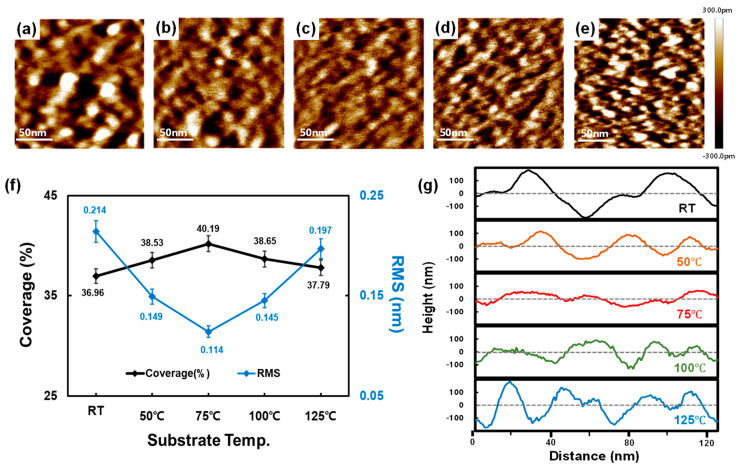
AFM image of V_2_O_5−x_ (5 nm) TF deposited at different substrate temperatures: (**a**) RT, (**b**) 50 °C, (**c**) 75 °C, (**d**) 100 °C, and (**e**) 125 °C. (**f**) Surface coverage and roughness (RMS) and (**g**) cross-sectional profile of each deposited V_2_O_5−x_ TF.

The AFM results (Figure 5) indicated that the deposited V_2_O_5−x_ of 5 nm thickness produced isolated islands. Before obtaining the AFM results, we actually expected that the surface coverage of these V_2_O_5−x_ islands would continue to increase with the substrate temperature because the temperatures at which we conducted the measurements were in the low-temperature regime [34,35,36]. However, as shown in Figure 5f, the surface coverage by V_2_O_5−x_ increased only until 75 °C, above which the islands again became more distinct. The uniformity of the V_2_O_5−x_ islands also followed this trend of coverage and revealed the lowest root mean square roughness (RMS) at 75 °C. Based on these AFM results, we confirmed that, even in this low-temperature regime, the morphology of the V_2_O_5−x_ TF was significantly affected.

**Figure 6:**
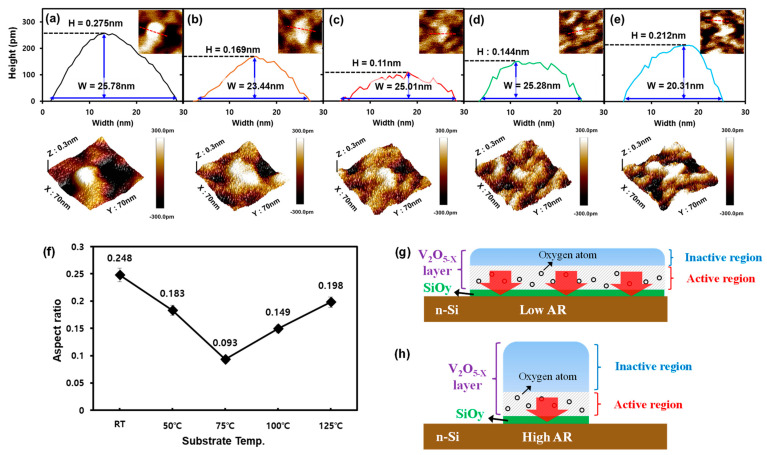
AFM images measured in 3D, and height and width of V_2_O_5−x_ islands at (**a**) RT, (**b**) 50 °C, (**c**) 75 °C, (**d**) 100 °C, and (**e**) 125 °C; (**f**) calculated aspect ratios (ARs). Schematic of the effect of the AR on the surface area of SiO_y_ for (**g**) low-AR and (**h**) high-AR V_2_O_5−x_ islands.

Based on this morphological sensitivity of the initial V_2_O_5−x_ islands to the deposition temperature, we surmised that the formation of the SiO_x_ passivation layer at the V_2_O_5−x_/Si interface was mainly influenced by the V_2_O_5−x_ morphology. To clarify our theory, we further investigated the change in the V_2_O_5−x_ morphology in terms of the aspect ratio (AR) of the initial V_2_O_5−x_ islands. Figure 6a–e shows the height (H) and width (W) of the V_2_O_5−x_ islands measured from the cross-sectional profiles of the AFM images to derive their AR at the different deposition temperatures. The obtained ARs are plotted in Figure 6f; this graph shows that the V_2_O_5−x_ islands formed at 75 °C had the lowest AR, 0.093, compared to those for the other temperatures. The advantage of such a low AR is that it can offer an expanded active region for supplying O atoms to the Si surface compared to those with higher ARs for the same amount of deposited V_2_O_5−x_ (Figure 6g,h). Therefore, a lower AR would be expected to result in the formation of a more effective SiO_y_ passivation layer over a larger Si surface resulting from V_2_O_5−x_ deposition.

**Figure 7:**
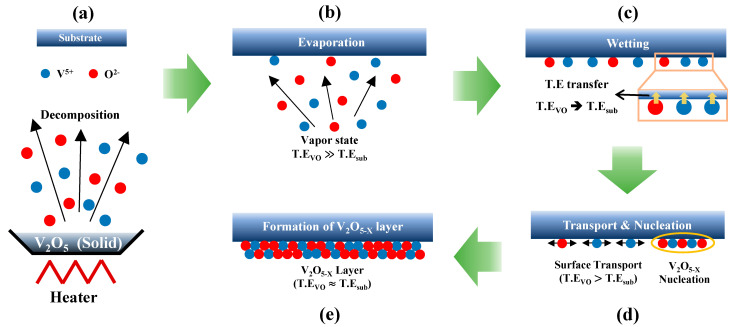
Schematic illustration of the solid-state V_2_O_5−x_ TF formation processes. (**a**) crucible heating (**b**) material evaporation (**c**) substrate wetting (**d**) surface transport and nucleation and (**e**) formation of V_2_O_5−x_ layer.

A possible mechanism whereby the thermally evaporated atoms are deposited is illustrated in Figure 7 as an attempt to explain the variation in the AR with the deposition temperature. Accordingly, the formation of the V_2_O_5−x_ TF occurs in five steps: (a) First, the solid-state V_2_O_5−x_ in the evaporation boat is decomposed into V and O atoms, respectively, in the vapor state during thermal deposition and (b) these atoms diffuse to the substrate. (c) As soon as the atoms reach the substrate, the substrate induces thermal energy (TE) loss, and the atoms are converted to the liquid state and wetted (or adsorbed) onto the substrate. (d) Then, the atoms are transported across the surface for nucleation of the V_2_O_5−x_ with the residual TE. (e) Finally, a continuous V_2_O_5−x_ TF is grown with the subsequent supply of V and O atoms [37,38].

**Figure 8:**
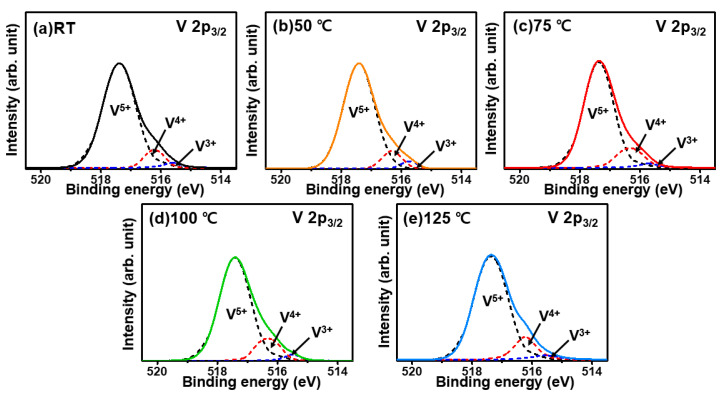
V 2p_3/2_ XPS profiles of V_2_O_5−x_ (15 nm) TFs deposited on Si surfaces at various deposition temperatures: (**a**) RT, (**b**) 50 °C, (**c**) 75 °C, (**d**) 100 °C, and (**e**) 125 °C.

**Figure 9:**
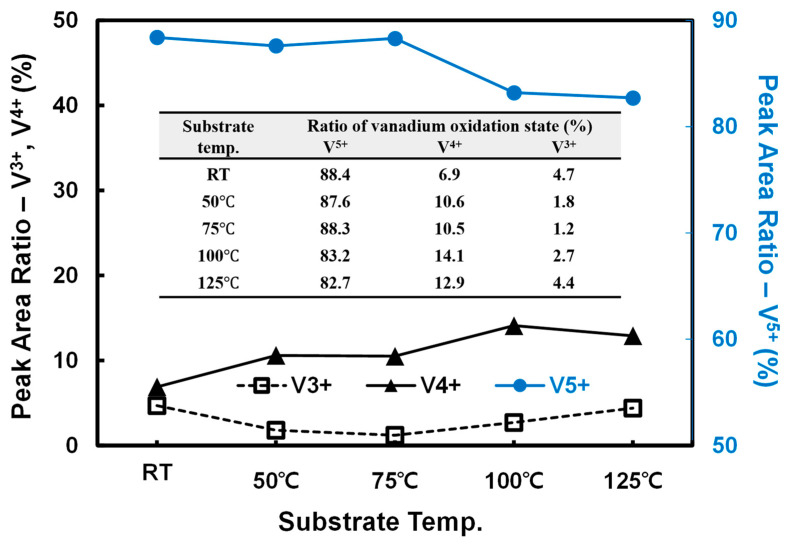
Integrated peak area ratios of vanadium oxidation states based on the measured V 2p XPS profiles.

In this process, assuming that the operating power, deposition distance, and vacuum conditions are the same, then the quality of the TF in terms of the stoichiometry, uniformity, and coverage would be most significantly affected by the distance the atoms are transported and the V_2_O_5−x_ nucleation rate on the surface in Figure 7d [39,40]. Based on this consideration, the observed AR variation with the deposition temperature can be explained by (1) the distance the atoms are transported and (2) nucleation rate. First, in terms of the transportation distance of the atoms, at low substrate temperatures, the amount of TE transferred from the atoms to the substrate would be relatively large to achieve thermal equilibrium between TE_VO_ and TE_sub_. This would result in the rapid adsorption of V and O atoms onto the surface when they reach the substrate, but the distance the atoms are transported would decrease owing to excessive TE loss. Therefore, at low substrate temperatures, the AR of the V_2_O_5−x_ islands would be expected to be high. In contrast, at higher substrate temperatures, TE transfer from the atoms to the substrate decreases. Hence, the depositing atoms could be transported for a comparatively longer distance, which would lower the AR of the V_2_O_5−x_ islands. However, as presented in Figure 6f, the AR of the V_2_O_5−x_ islands gradually decreased only until 75 °C, after which it increased again at higher temperatures (i.e., 100 and 125 °C). On the basis of this behavior, we assume that the nucleation rate for the V_2_O_5−x_ would decrease at temperatures above 75 °C, at which more metallic V-rich V_2_O_5−x_ islands would form to increase the AR [40,41]. To confirm this assumption, additional XPS measurements were performed to investigate the stoichiometry of the V_2_O_5−x_ TFs deposited at different temperatures and the results are presented in Figure 8 and Figure 9. The results confirmed that deposition temperature notably affected the composition of the vanadium oxidation states (VOS) in the deposited V_2_O_5−x_ TFs.

In Figure 8, the V 2p XPS results exhibit no change in the peak positions at the tested temperatures; the peaks are assigned to V_2_O_5−x_. Figure 8 shows that the deposition of V_2_O_5−x_ on Si occurs via the formation of various VOS, identified as V^5+^ with a high binding energy, and V^4+^ and V^3+^ with lower binding energies, respectively [7,42,43]. The measured XPS profiles were deconvoluted to distinguish the individual VOS within the V_2_O_5−x_ TFs, followed by calculation of the peak areas to compare the composition ratios of the three VOS, namely, V^5+^, V^4+^, and V^3+^ (Figure 9). The results from the Figure 9 showed that, as the deposition temperature increases from RT to 75 °C, the ratio of V^5+^ is preserved at approximately 88%, whereas that of V^4+^ increases from 6.9% to 10.5%. With respect to V^3+^, the increase in the V^4+^ state likely arises from the decrease in the ratio of the V^3+^ state from 4.7% at RT to 1.2% at 75 °C, seemingly as a result of the oxidation of V^3+^ to V^4+^.

However, as the deposition temperature increases to 100 °C, the V^5+^ state, which remained at approximately 88% of the VOS up to 75 °C, sharply decreased to 83% at higher temperatures. Simultaneously, the ratio of V^4+^ increased from 10.5% to 14.1%, and that of V^3+^, from 1.2% to 2.7%. Thus, the VOS in the TF underwent reduction to lower states. This trend was more pronounced when the deposition temperature was increased to 125 °C. This implies that the nucleation of V_2_O_5−x_ above 75 °C is limited because of its high temperature [40]. Therefore, as indicated in Figure 9 and the embedded table, the ratio of lower VOS would increase as a result of the significant loss of volatile O atoms from the surface followed by the formation of V_2_O_5−x_ islands with high AR, as shown in Figure 6.

With these results, we demonstrated that the optimal deposition temperature can promote the passivation capability of the deposited V_2_O_5−x_ TF owing to the effective formation of the SiO_y_ layer at the V_2_O_5−x_/Si interface because of the low AR of the initial V_2_O_5−x_ islands. However, this facilitated formation of SiO_y_ could also improve the electrical conductivity of the V_2_O_5−x_ TF itself by inducing the generation of O deficiencies in the V_2_O_5−x_ TF [25,44]. Therefore, using the TLM, the electrical conductivities were also measured as a function of the deposition temperature, and the results are shown in Figure S1. The electrical conductivities measured above 75 °C were higher compared to those at lower temperatures and this was mostly attributed to the higher occurrence of lower VOS within the V_2_O_5−x_ TFs above 75 °C.

**Figure 10:**
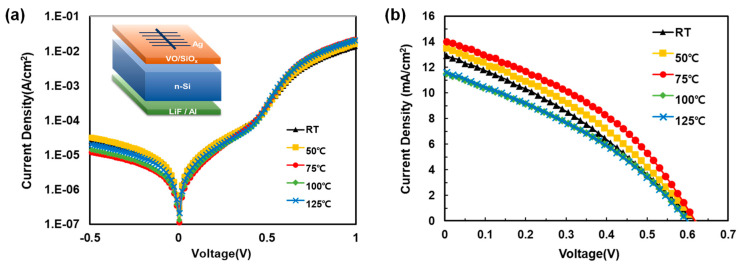
Current density vs. voltage curves of Si/V_2_O_5−x_ HSCs measured under (**a**) dark and (**b**) 100 mW/cm^2^ illumination (AM1.5) conditions.

Finally, the relationship between the V_2_O_5−x_ deposition temperature and the performance of the solar cell was investigated by fabricating Si/V_2_O_5−x_ HSCs at different substrate temperatures, as shown in Figure 10. Figure 8b shows the current density vs. voltage (J–V) curve for the fabricated HSCs, and Table 1 summarizes the HSC performance parameters. These results clearly confirmed that the HSC fabricated at 75 °C had the highest PCE value of 3.3%, compared to those fabricated at the other temperatures. As is clear from Table 1, the overall solar cell performance of the HSC fabricated at 75 °C is higher compared to that at the other temperatures. Most notably, in terms of FF, the 75 °C sample had the highest value of 37.8%. For the Si-based solar cell, effective passivation on high defect states on the surface is crucial to improve R_sh_. In addition, providing reduced sheet resistance of contact layers is also highly important to decrease R_s_. Therefore, as shown in Figure 2 and Figure S1, the enhanced passivation effect at 75 °C and improved V_2_O_5−x_ conductivity were the main factors for the improve PCE with the 75 °C sample [45,46]. However, the morphology of the V_2_O_5−x_ TF presumably also affected the value of R_s_ considering that the smoothest surface morphology was produced at 75 °C, thereby improving the metal contact resistance in the HSCs.

## 4. Conclusions

In this report, we presented our systematic comparative study to explore the effect of the deposition temperature on the ability of the V_2_O_5−x_ TF to passivate the Si surface. Our results confirmed that the $\tau_{eff}$ can be considerably enhanced even with a minor elevation in the temperature at which the V_2_O_5−x_ TF is deposited below 100 °C. The XPS measurements showed that formation of the SiO_x_ passivation layer at the Si/V_2_O_5−x_ interface is highly facilitated even at a temperature as low as 75 °C, which is an unprecedentedly low temperature for TMO thermal treatment in general. To investigate the origin of the improved passivation effect at such a low temperature, the morphologies of the initial V_2_O_5−x_ islands were analyzed. The AR of the V_2_O_5−x_ islands was found to be highly sensitive to even minor changes in the deposition temperature below 125 °C. In addition, a detailed study of the VOS with XPS revealed that the stoichiometry of the V_2_O_5−x_ TF was also notably affected by the deposition temperature. Consequently, a specific temperature, 75 °C, was found to produce V_2_O_5−x_ islands with the lowest AR, which offers the most-expanded V_2_O_5−x_-active region to supply O atoms to the Si surface for effective SiO_y_ layer formation. As a result, the PCE of fabricated Si/V_2_O_5−x_ HSCs was noticeably higher at our optimized deposition temperature compared to the PCE of the other HSCs.

## Figures and Tables

**Table 1:** Solar cell performance parameters of Si/V_2_O_5−x_ HSCs.

Substrate Temp.	J_sc_ (mA/cm^2^)	V_oc_ (mV)	FF (%)	R_sh_ (Ω·cm^2^)	R_s_ (Ω·cm^2^)	PCE (%)
RT	12.98	618	32.85	13027	7.20	2.63
50 °C	13.51	619	34.72	13574	6.01	2.90
75 °C	14.09	618	37.81	14122	5.65	3.29
100 °C	11.54	601	34.63	11583	7.18	2.40
125 °C	11.64	600	34.23	11693	7.60	2.39

## Supplementary Materials

The following supporting information can be downloaded at: https://www.mdpi.com/article/10.3390/ma15155243/s1, Figure S1: (a) Measured sheet resistance of VO TFs according to Si substrate temperature extracted from the ohmic current-voltage response in TLM measurements and (b) derived conductivities of each VO TFs; Figure S2: Experimental Mott-Schottky plots and linear fittings for the samples from the different deposition temperature. Reference [47] are cited in the supplementary materials.
