# Supplementary material for: Stoichiometry and Morphology Analysis of Thermally Deposited V2O5−x Thin Films for Si/V2O5−x Heterojunction Solar Cell Applications
Source: Materials (Basel). 2022 Jul 29;15(15):5243. doi: 10.3390/ma15155243 (PMC9369836; doi:10.3390/ma15155243)
Supplement: Supplementary file 1 [file materials-15-05243-s001.zip › materials-1829685-supplementary.pdf]

Supporting Information

Stoichiometry and Morphology Analysis of Thermally Deposited V_2O_{5-x} Thin Films for Si/V_2O_{5-x} Heterojunction Solar Cell Applications

Gwan Seung Jeong [1], Yoon-Chae Jung [1], Na Yeon Park [1], Young-Jin Yu [1], Jin Hee Lee [2], Jung Hwa Seo [3] and Jea-Young Choi [4,*]

[1] Department of Metallurgical Engineering, Dong-A University, Busan 49315, Korea; wjdrhkstmd12@gmail.com (G.S.J.); dbsco0306@naver.com (Y.-C.J.); nayeon2385@gmail.com (N.Y.P.); tq6284@naver.com (Y.-J.Y.)
[2] Department of Chemical Engineering, Dong-A University, Busan 49315, Korea; dljh82@gmail.com
[3] Department of Physics, University of Seoul, Seoul 02504, Korea; seojh@uos.ac.kr
[4] Department of Materials Sciences & Engineering, Dong-A University, Busan 49315, Korea
* Correspondence: cjy4395@dau.ac.kr

In order to obtain the electrical conductivity of the deposited VO TF according to the Si substrate temperature, Transmission-Line-Method(TLM) measurement were performed for the samples fabricated in Ag(200nm)/VO(15nm)/n-Si structure. In Figure 2, the results of TLM measurement and the derived electrical conductivity after considering VO TF thickness are provided. From the results, we confirmed that 75°C can provide the most improved electrical conductivity compared to those from other temperature condition. However, the overall electrical conductivity was sharply increased from 75°C which was mainly attributed by the increased ratio of lower VOS within VO TF.

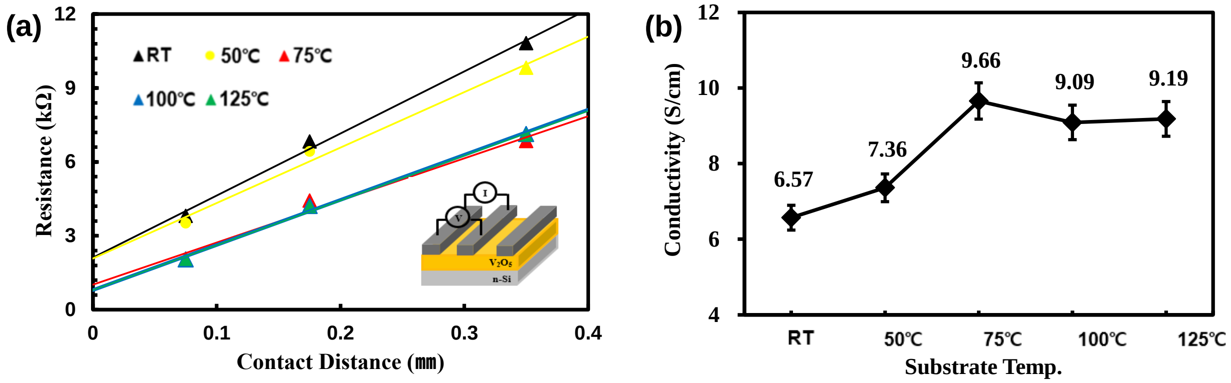

Figure S1. (a) Measured sheet resistance of VO TFs according to Si substrate temperature extracted from the ohmic current-voltage response in TLM measurements and (b) derived conductivities of each VO TFs.

In addition, Capacitance-Voltage(C-V) characteristics were also measured for the samples with different VO deposition temperatures which were fabricated in Ag(200nm)/VO(15nm)/n-Si/Al(200nm) structure. From the C-V measurement, we induced built-in potential (V_{bi}) formed at Si/VO interface and the results are shown in Figure S2. As shown in the inlet graph, sample from 75°C revealed the highest V_{bi} value of 0.82 V. This increased V_{bi} at 75°C can mainly attributed to improved stoichiometric of VO TFs.[1]

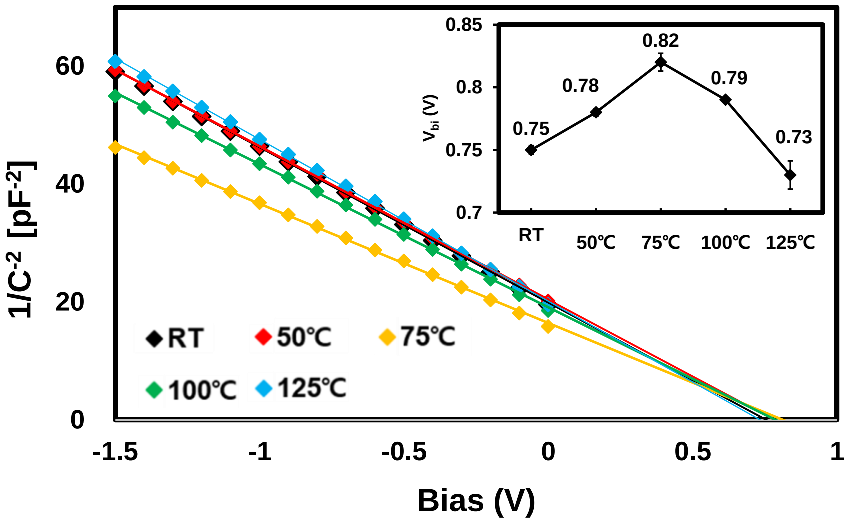

Figure S2. Experimental Mott-Schottky plots and linear fittings for the samples from the different deposition temperature.

References

1. Almora, O.; Gerling, L.G.; Voz, C.; Alcubilla, R.; Puigdollers, J.; Garcia-Belmonte, G._Superior performance of V2O5 as hole selective contact over other transition metal oxides in silicon heterojunction solar cells. *Sol. Energy Mater. Sol. Cells* **2017**, *168*, 221–226.
